# The Tuberculosis Danger among Recruits

**Published:** 1914-10-17

**Authors:** 


### THE TUBERCULOSIS DANGER AMONG RECRUITS.

We publish in our Notes this week a report made by Dr. Symons, the Medical Officer of Health of Bath, upon the presence of undetected cases of phthisis amongst recently enrolled recruits. The facts, for which he vouches as the result of personal investigation, are startling, and raise the question as to the efficiency of the medical examination to which applicants for enlistment are submitted before acceptance. There is a hint in the wording of Dr. Symons' report that the failure to detect these cases is, in his opinion, partly due to the fact that the medical examiners are mostly general practitioners. We think that this insinuation, natural to the specialist, must not be taken for more than it is worth. In advanced cases (for such Dr. Symons seems to have in mind), if they could not diagnose phthisis, it would be obvious that there is something radically wrong with our system of medical education and the conditions requisite for qualification. We suspect that the real reason is to be found, as Dr. Symons also suggests, in the conditions under which the examination of recruits is carried on.

As everyone knows, the number of applicants to join the Army has been immense, and the provision of medical men to examine them has been most inadequate. When the pressure was at its highest men had to wait for hours to be examined, and often had to return day after day before they could be attended to. Moreover, the unavoidable noise and confusion of a busy recruiting station must interfere materially with the recognition of the slight auscultatory and percussion changes upon which a diagnosis of the earlier stages of phthisis depends. Taking into consideration also the natural tendency of an eager candidate to suppress information as to his symptoms which might lead to his rejection, it is not, after all, so great a matter for surprise that some cases which ought to have been rejected should have been accepted. We think, too, that Dr. Symons is rather hard upon the enthusiastic candidate for the Army when he states that his failure to give the examiner the full details of any symptoms from which he may have suffered is almost "criminal." We agree that, as would appear to have been the case in one instance cited, any man who suppressed the fact that he had been under treatment for phthisis would be deserving of the severest reprobation. A case like this is, however, probably quite exceptional. In the absence of evidence to the contrary, we would rather believe that in the majority of cases eagerness to serve his country has led the candidate to make little of symptoms which, under ordinary circumstances, he would have divulged fully.

The blame for the failure of the medical examination, revealed by these investigations at Bath, lies, we think, at the doors of the military authorities. Faced by an unprecedented rush of candidates for enlistment, they failed to realise the necessity of a stringent medical test, and attached too much importance to a standard of simple physical measurements. A healthy man of 5 ft. 3 in. would make a better soldier than a man of 6 ft. with early phthisis or other progressive disabling disease presenting slight physical signs, yet the former would be rejected on sight, whilst the latter might easily pass a hurried medical examination, only to break down in the early days of his training. But there is no real reason why the medical examination should be a hurried one. If the military authorities had asked for volunteer helpers, they could have obtained as many medical men as they wanted to assist the Army doctors in their examination of recruits. When such assistance was asked for it was at once forthcoming, but in many cases offers of help of this kind met with no acceptance.

The matter is of the greater importance because the presence of these unrecognised cases of phthisis is a real source of danger. The conditions under which recruits have to be housed in crowded barracks, temporary huts, and small tents, not to speak of the close contact in the trenches in actual warfare, present the fullest opportunities for the conveyance of tubercular infection. There should be no necessity to dwell upon this risk. It must be obvious to every medical man and to every educated layman. The remedy is equally obvious: a medical examination sufficiently stringent to detect even the earliest cases. But such an examination would require a considerable increase in the number of examiners. This increase involves no difficulties. The military authorities have only to ask, and there is hardly a medical man in the United Kingdom who would not be willing to devote an hour or two daily to the task. For the sake of this important moral we have taken the above investigation at its face value.

